# Primary pleomorphic liposarcoma involving bilateral ovaries: Case report and literature review

**DOI:** 10.1515/biol-2025-1068

**Published:** 2025-03-21

**Authors:** Huan Chen, Jing Luo, Ke Zhang, Puxiang Chen

**Affiliations:** Department of Obstetrics and Gynecology, Zhuzhou Central Hospital, Zhuzhou, 412000, China; Department of Obstetrics and Gynecology, The First People’s Hospital of Jinghong, Jinghong, 666100, China; Department of Obstetrics and Gynecology, The Second XIANGYA Hospital of Central South University, Changsha, 410000, China; Department of Pathology, The Second XIANGYA Hospital of Central South University, Changsha, 410000, China

**Keywords:** ovarian cancer, pleomorphic liposarcoma, ovary, case report, sarcoma

## Abstract

Pleomorphic liposarcomas (PLPSs) commonly occur in the extremities or retroperitoneum. However, cases of primary PLPSs in the female reproductive system are rare, with only one reported case in the ovary. Herein, we describe the case of a patient with primary PLPS involving bilateral ovaries. She presented with a 2-month history of abdominal pain, and underwent total hysterectomy with bilateral salpingo-oophorectomy, omentectomy, and excision of surface lesions on the bladder and rectum. Intraoperatively, nitrogen mustard (2%) was used to rinse the abdominal cavity. One week postoperatively, she was administered the first cycle of postoperative cisplatin chemotherapy for intraperitoneal heat infusion chemotherapy plus intravenous liposomal paclitaxel. Postoperative histopathological examination revealed primary PLPS involving both ovaries. Therefore, a doxorubicin liposomal chemotherapy regimen was administered according to the soft tissue sarcoma NCCN guidelines. The patient’s CA-125 levels decreased from 987 to 9.8 U/mL; however, after two chemotherapy sessions, she declined further treatment. The patient was still being followed-up and had no signs of recurrence at the time of writing this report. PLPS tends to be misdiagnosed and underdiagnosed due to its complex pathology and the lack of specific molecular markers. The disease is infrequent in the female reproductive system, and there is no consensus on its diagnostic and therapeutic guidelines. Herein, we summarized the findings of published case reports of PLPSs in organs of the female reproductive system to raise awareness of the disease and discussed its diagnosis, clinical treatment, and prognosis.

## 1 Introduction

Soft tissue sarcoma (STS) is a malignant neoplasm that can occur anywhere in the body, including adipose, muscular, and fibrous tissues, as well as blood vessels [1]. Liposarcomas (LPSs) account for approximately 20% of all sarcomas [2]. According to the 2020 WHO classification, LPSs are classified into six types: well-differentiated and not otherwise specified LPSs (lipoma-like, inflammatory, and sclerosing), dedifferentiated LPS, myxoid LPS, pleomorphic LPS (PLPS), epithelioid LPS, and myxoid PLPS [3]. PLPS is a rare aggressive variant that accounts for only 5% of cases [4]. The extremities are the most common sites for PLPS (60%), followed by the chest and abdominal walls (15%), the retroperitoneum (5%), and other anatomical sites (20% in total). The local recurrence rate of PLPS is 30–45%, its metastatic rate is 28–43%, and the reported disease-specific mortality ranges from 28 to 35%. PLPSs metastasize most commonly to the lungs (75% of cases) and liver (25% of cases). An increased tumor size, a high mitotic rate, truncal and deep locations, and vascular invasion are associated with a poor prognosis [5]. Few case reports have focused on PLPS occurring in the female reproductive system. The pathogenesis, presentation, surgical treatment, and postoperative features of this disease remain unclear. Herein we present the case of a patient with PLPS involving both ovaries, who was treated with hyperthermic intraperitoneal chemotherapy. To our knowledge, this is the first reported case in medical literature.

## 2 Case representation

A 55-year-old postmenopausal woman (para 1) was referred to our hospital with a 2-month history of abdominal pain and unintentional weight loss of approximately 10 kg. She had a 16-year history of schizophrenia and was treated with antipsychotropic medications for several years. Her family history was significant for symptomatic uterine fibroid in her mother and two elder sisters, and her father had died of pancreatic cancer.

**Figure 1:**
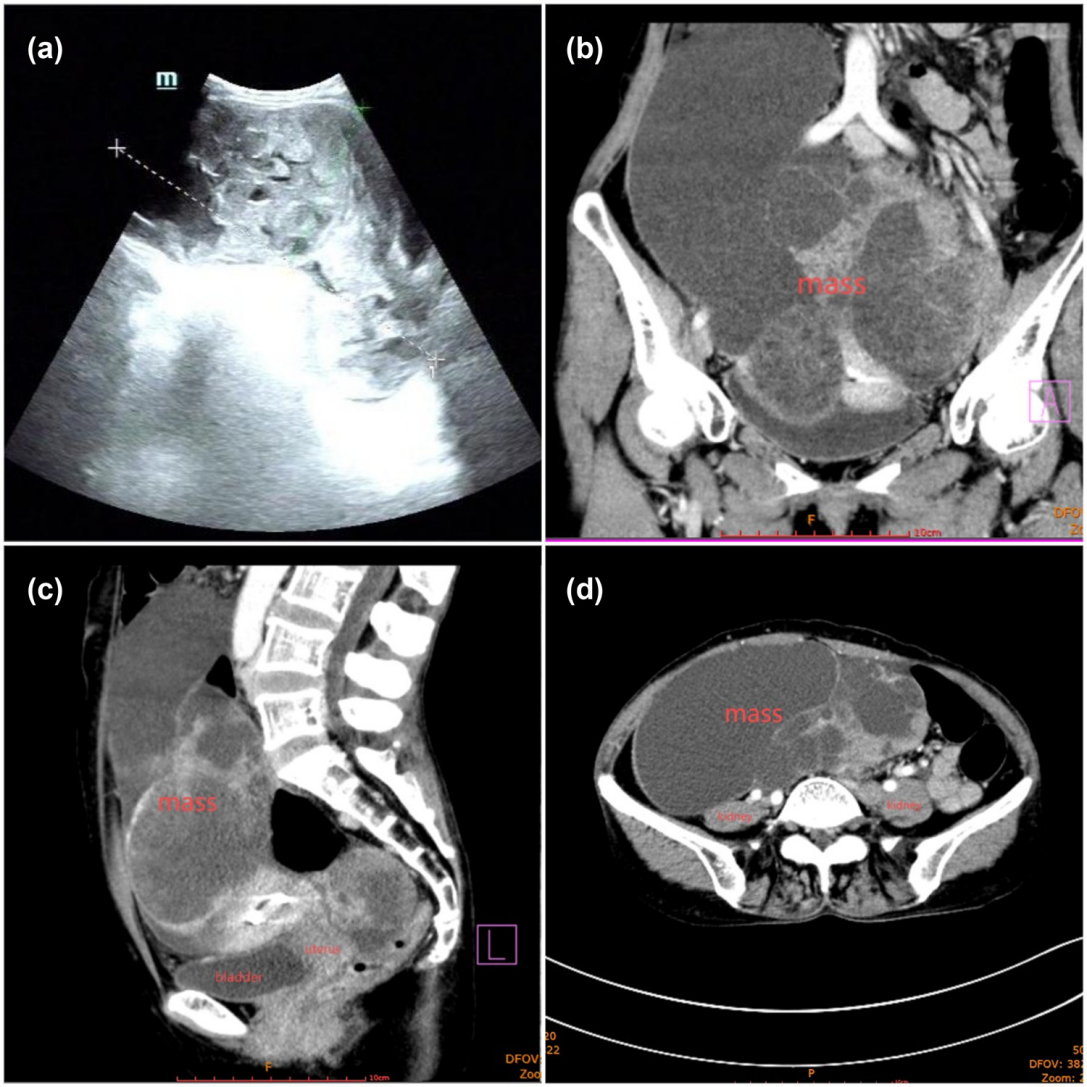
B-ultrasound and CT image of the patient. (a) B-ultrasound showed a multilocular, mixed solid, and cystic mass. (b)–(d) CT scan revealed a 133 mm × 229 mm mass located in the pelvic and abdominal cavity; it was closely adhered to the uterus, the upper bladder wall, and the anterior rectal wall, with unclear demarcation. The mass was solid and cystic, with septations and obvious enhancement; there were no signs of pelvic effusion, and no swollen lymph nodes were observed in the abdominopelvic cavity.

On admission, her vital parameters were within normal limits. A physical examination revealed a medium-textured lower abdominal mass extending two fingerbreadths above the umbilicus; the rest of the examination revealed no abnormalities. B-ultrasound showed a multilocular, mixed, solid, and cystic mass (Figure 1a), consistent with an ovarian origin tumor. Computed tomography (CT) scan revealed a 133 mm × 229 mm mass spanning the pelvic and abdominal cavities and closely adherent to the uterus, the upper bladder wall, and the anterior rectal wall, with unclear demarcations. There were no signs of pelvic effusion or lymphadenopathy in the abdominopelvic cavity (Figure 1b). CT of other parts of the body revealed no sign of oncological involvement. However, the patient’s CA-125 levels were elevated at 987 U/mL, while her HE4 levels were within normal limits. Fibrogastroscopy findings were indicative of chronic gastritis.

We suspected she had a clinically advanced ovarian cancer (OC) due to the pelvic mass on pelvic CT and ultrasound, along with the significant increase in CA125 levels. The patient then underwent an exploratory laparotomy. Intraoperatively, approximately 1,000 mL of yellowish ascitic fluid was found in the abdominal cavity; no abnormalities were observed in the liver, gallbladder, spleen, kidneys, stomach, diaphragm, and small intestine. The mass was (20 cm) mixed cystic and solid, brittle; the solid part of the mass had tofu brain-like changes, with evidence of invasion of the lower part of the descending colon, the entire length of the sigmoid colon, and the plasma membrane of the upper part of the rectum; the right fallopian tube was invaded by the mass and structurally destroyed; the peritoneum over the right pelvic wall was also invaded and thickened. The uterus was in a posterior position; the size and texture of the uterus were grossly normal; however, its surface had multiple miliary nodules. The right ovary appeared only slightly enlarged with a smooth outer surface. The peritoneal covering of the bladder was invaded by the mass, with diffuse thickening and an obvious metastatic mass measuring 6 cm × 5 cm. The ischiorectal fossa had multiple metastatic miliary nodules, while no nodules were observed in the paracolic grooves. A frozen-section biopsy revealed the mass malignancy. The patient was diagnosed with stage Ⅲc OC intraoperatively; consequently, the surgeon performed a total hysterectomy, left salpingo-oophorectomy, and omentectomy. Furthermore, both the bladder and rectal surface lesions were all meticulously excised. Lymph node dissection was not performed due to considerations of late staging and the implications on quality of life. During the operation, nitrogen mustard (2%) was used to rinse the abdominal cavity. The patient had an uneventful recovery. One week postoperatively, hyperthermic intraperitoneal cisplatin (110 mg) chemotherapy plus intravenous liposomal paclitaxel (175 mg/m^2^) chemotherapy were administered once while awaiting the pathology results. After this cycle of chemotherapy, the patient’s CA-125 level reduced to 21.1 U/mL. One month later, the pathology suggests the presence of PLPS in both ovaries, as well as the bladder and rectal surface lesions; however, the omentum and ascitic fluid were free of tumor cells. The chemotherapy regimen was then changed to liposomal doxorubicin (2 mg/kg). The patient’s CA-125 level dropped further to 9.8 U/mL after the second chemotherapy. The patient had no complaints during chemotherapy sessions; however, for personal reasons, she declined further treatment. At the time of writing this report, the patient had been under periodic follow-up examinations and had had no signs of recurrence for 22 months. Further CT scans revealed no abnormalities, and her CA125 level was 7.52 U/mL.

The gross specimen of the left ovarian mass was approximately 20 cm × 14 cm × 8 cm in size. It appeared multilocular and exhibited a combination of cystic, solid, and partly solid areas with a tofu brain-like texture. The mass was friable and contained yellow mucus-like material inside the capsule. The right ovary had the same features as the left one upon dissection.

**Figure 2:**
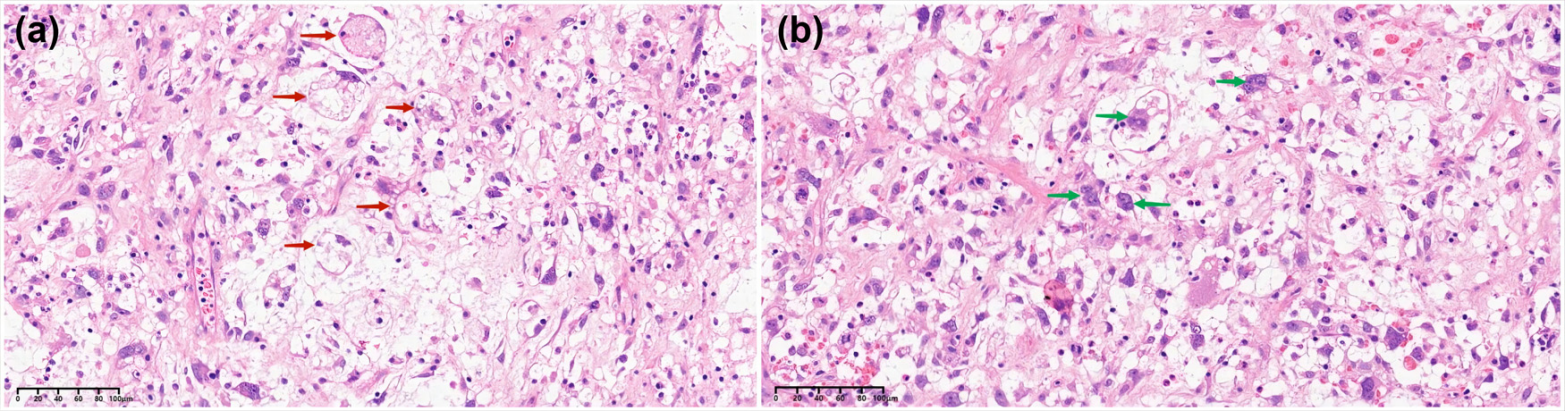
Pathology image of the patient. (a) HE showed rounded, irregular, and signet-ring adipoblasts, as well as typical pleomorphic adipoblasts with large nuclei and deeply stained nuclei (red arrow). (b) Multivacuolar changes in the interstitium were seen in areas of lipogenic origin; atypical multinucleated giant cells of various shapes with eosinophilic or vacuolated cytoplasm are seen in non-lipogenic areas (green arrow).

The pathological findings of both ovaries revealed rounded, irregular, and signet-ring adipoblasts, as well as typical pleomorphic adipoblasts with large and deeply stained nuclei, along with multivacuolar interstitial changes in lipogenic origin areas, without normal ovarian tissues (Figure 2). The non-lipogenic areas contained atypical multinucleated giant cells of various shapes with eosinophilic or vacuolated cytoplasms. In addition, malignant neoplastic components were found in both the bladder and rectal surface lesions.

Immunohistochemistry showed positive staining for P53 (70%+), vimentin, Ki-67 (80%+), and CD99, and partial staining for CDK4. The results were negative for S100 and MDM2.

Next-generation sequencing analysis results revealed five somatic mutations, including *TP53 p.S241F*, *ARAF p.V143F*, APC p.T185Ffs*4, *EPHB1 p.D848E*, and *ABL2 p.P986L*. The tumor mutational burden was 3.84 mutations/Mb. A microsatellite instability analysis revealed microsatellite stability. No germline variants were found; however, the following genes were fused *AKAP8L-BRD4(EX2:EX2)*, *FBXL7-FAM105B(EX1:EX3)*, and *CYP7B1-UCKL1(EX1:EX2)*.


**Informed consent:** Informed consent has been obtained from all individuals included in this study.
**Ethical approval:** The research related to human use has been complied with all the relevant national regulations, institutional policies and in accordance with the tenets of the Helsinki Declaration, and has been approved by the Ethics Committee of the Second Xiangya Hospital affiliated to Central South University.

## 3 Discussion

The gold standard for diagnosing PLPS is the presence of polymorphic lipoblasts [6]. However, polymorphic lipoblasts need to be distinguished from the “homogeneous lipoblastoid differentiation” cell types seen in dedifferentiated LPSs. Differentiating PLPSs from other high-grade sarcomas or high-grade carcinomas may be difficult due to the complex morphology of these tumors. PLPS tumors exhibit variable distribution of pleomorphic lipoblasts in different regions, and identifying them poses a challenge. Moreover, the tumor morphologies vary between cell-rich pleomorphic sarcomatoid, epithelioid, muco-fibrosarcomatoid, and scattered foci of coagulomatous necrosis [7]. Ultrasound PLPS features exhibit a mixed hyperechoic and hypoechoic pattern reminiscent of gyri [8]; the ultrasound images of our patient had such an appearance. The molecular features of PLPSs include 13q14.2-5 (containing *RB1*) deletions in 50% of patients, *TP53* mutations, and *NF1* deletions [5,6]. Our patient had mutations in other genes in addition to the TP53 mutation. However, the p53 mutation is not seen in other LPS subtypes and is considered to be the cause of chemoresistance in PLPS [9].

According to the STS NCCN guidelines [10], a wide surgical resection with negative tumor margins is a potentially curative strategy for non-metastatic primary retroperitoneal or intraperitoneal sarcomas (R0 resection/R1 resection recommendation). After surgery, adjuvant systemic therapy, including radiation and chemotherapy, should be considered for all patients with metastatic disease risks based on intraoperative or clinicopathological findings. However, the expert panel discouraged adjuvant radiation therapy (RT) for retroperitoneal/intra-abdominal STSs except for highly selected cases, in which local recurrence would cause undue morbidity. A previous study showed that R1 resection was associated with a two-fold higher risk of recurrence, while adjuvant chemotherapy appeared to improve the relapse-free survival (RFS) in patients with STSs of the extremities. Postoperative doxorubicin-based chemotherapy significantly improved recurrence-free survival and overall survival in female patients with R1 resection. Although the mass was excised to the extent that it was invisible to the naked eye intraoperatively, we could not guarantee that there would be no microscopic residue and believe that the resection only reached R1. Therefore, we administered postoperative adjuvant chemotherapy to the patient and changed the regime according to the routine pathology. However, the evidence regarding whether chemotherapy is beneficial for overall patient survival is conflicting [10]. Moreover, due to its low incidence, there is no evidence on chemotherapy being beneficial for survival in women with PLPS in the female reproductive system.

Only a few cases of PLPS originating in the female reproductive system have been reported. The pathogenesis, surgical treatment, and postoperative features of PLPS remain unclear. A search of the PubMed, Embase, WOS, and CNKI databases yielded six cases of PLPS in the uterus [11–16], two in the cervix [17,18], one in the perineal corpus [19], one in the fallopian tube [20], and one in the ovary [21]. Table 1 summarizes all published PLPS cases in the female reproductive system: the patients age ranged from 24 to 70 years, with a median age of 50.8 years; the presenting symptoms included abdominal pain, dysuria, urinary retention, anorexia, increasing abdominal girth, vaginal bleeding, uterine mass, and a perineal lump. Most patients underwent surgical resections, radical/total hysterectomy along with bilateral adnexectomy, with or without lymphadenectomy. Adjuvant therapy included chemotherapy, RT, and targeted therapy. Most patients tested positive for S-100 and vimentin; however, our patient was only positive for vimentin. Negative expressions of MDM2 and CDK4 have been proposed as markers for PLPS from well-differentiated LPSs and dedifferentiated LPSs [22]; however, CDK4 was partially positive and MDM2 was negative in our case.

**Table 1:** The clinical character of all primary PLPS originated from female genital system

References	Year	Site	Age	symptom	Size	Surgery	IHC	NGS	Adjuvant therapy	Recurrence	Follow up
Valenciaga [11]	2021	Uterus	70	Acute abdominal pain	7.0 × 6.0 × 2.1 cm(uterine anterior) 8.0 × 6.5 × 4.0 cm(uterine posterior)	TAH + BSO(first) right PH(recurrence)	CD10 cyclin-D1	IQGAP-NTRK3	C(doxorubicin) + T(olaratumab), T(Entrectinib live recurrence), R(neck recurrence)	15 m (liver), 18 m (neck)	49 m
Schoolmeester [12]	2016	Uterus	70	Dysuria and urinary retention	9.0 × 8.0 × 7.5 cm	RH + BSO + PLND	S-100	TP53, PTEN, RB1, FAT1, TERT	C(gemcitabine + docetaxel)		Died, One circle, chemotherapy
McDonald [13]	2011	Uterus	49	Uterine mass	10.5 cm	TAH + BSO + appendectomy	S-100, MDM2	Not, Report	none	no	12 m
Fadare [14]	2011	Uterus	62	Acute abdominal pain	7 × 6.3 × 4.5 cm	TAH + BSO + reimplantation of a resected left ureter with stent placement	Not report	Not Report	C(gemcitabine + taxotere) after resction of recurrent lesion	2 m anterior abdominal wall	Lost
						Nodule of anterior abdominal wall resection(recurrence)					
Levine [15]	2003	Uterus	62	Lower abdominal pain, anorexia, and increasing abdominal girth	15 cm	RH + BSO + PLND paraaortic lymph node dissection, and omentectomy	S-100 vimentin ER	Not Report	R after recurrence	9 m multiple pelvic nodules	Not report
Sośnik [16]	2006	Uterus	71	Vaginal bleed ing	10.8 × 12.9 × 6.0 cm	TAH + BSO	S-100	Not Report	R	No	96 m
Tandon [17]	2017	Cervix	24	None	6 cm	None	S-100	TP53,ALK	C(cisplatin + Etoposide)	No report	died shortly
Obafunwa [18]	1990	Cervix	45	Not available	9 cm	Not available	Not available	Not available	Not available	6 m	Not available
Gondos [19]	1982	Perineum	31	Perineal lump	6 × 5 × 4 cm	TPE + perineum + vulva + posterolateral vagina resection(first)	Not report	Not report	C(Adriamycin)after recurrence	8 m	14 m
						Resection of left vagina(recurrence)					
Wang [20]	2017	Fallopian Tube	47	Dull pain in the left lower quadrants of the abdomen	10 × 10 × 9 cm	TAH + BSO	Ki-67	Not report	C(Ifosfamide + epirubicin)	10 m Left lower quadrants of the abdomen	11 m
Gao [21]	2013	Left ovary	28	Lower abdominal pain	10 cm	Left salpingo-oopherectomy	S-100 vimentin	Not report	C	No	96 m

TPE: Total pelvic exenteration; RH, radical hysterectomy; TAH, total hysterectomy; BSO, bilateral salpingo-oopherectomy; PLND, pelvic lymph node dissection; PH: partial hepatectomy; C, chemotherapy; R, radiotherapy; T: Targeted therapy; IHC: Immunohistochemistry; NGS: Next generation sequencing.

No standard treatments exist for LPSs occurring at rare sites, including those in the female reproductive system. Among the treatment modalities mentioned in the 11 reported cases we retrieved, one patient underwent surgery, chemotherapy, radiotherapy, and targeted therapy; another one received only surgical treatment; two patients received surgery and radiotherapy; five patients received surgery and chemotherapy; one patient received only chemotherapy; however, we could not ascertain the treatment information for the other patient. Overall, surgery combined with adjuvant therapy seems to be the mainstream treatment approach, and patients who received chemotherapy alone have a poor prognosis. Although adjuvant RT following surgery is discouraged for retroperitoneal or intra-abdominal sarcomas, two of the patients in the published case reports received adjuvant radiotherapy. Most of the women in the reports received chemotherapy, but only three underwent the preferred chemotherapy regimen based on the NCCN guidelines for STS (adriamycin, ifosfamide, and epirubicin); the remaining patients received other recommended NCCN chemotherapy regimens for recurrent OC (such as cisplatin and etoposide, gemcitabine and docetaxel, or gemcitabine and taxotere). In the case of our patient, the first chemotherapy regimen was based on the NCCN guidelines for OC [23], while awaiting the results of the pathological examination. Once the pathology results were consistent with PLPS, the chemotherapy regimen was replaced with doxorubicin. The evidence showing that postoperative chemotherapy improves the RFS of patients is based on patients with STSs of the extremities [10]. Therefore, whether postoperative chemotherapy is beneficial for patients with PLPS of organs of the female reproductive system remains unclear. Doxorubicin alone or in combination with ifosfamide is the preferred choice for most STSs; however, different LPS subtypes have different sensitivities to chemotherapeutic agents [24], and most studies have been conducted with patients with PLPSs at frequently occurring sites. Therefore, whether these drugs are effective for PLPS in rare sites is unknown. Our patient was free from relapses 22 months after stopping the treatment. Therefore, we speculate that intraperitoneal heat-perfusion chemotherapy may be equally effective in ovarian malignancies of mesenchymal origin, with some clinical implications. For advanced PLPS, eribulin and trabectedin have shown promising activity in comparison to conventional therapy (doxorubicin- and gemcitabine-based regimes) [25].

In conclusion, prospective studies with relatively large samples are needed to determine whether the surgical approach and postoperative treatment for PLPSs originating in the female reproductive system should be managed according to STS or corresponding malignant tumor guidelines for the female reproductive system.
